# Differential regulation of collapsin response mediator protein 2 (CRMP2) phosphorylation by GSK3ß and CDK5 following traumatic brain injury

**DOI:** 10.3389/fncel.2014.00135

**Published:** 2014-05-28

**Authors:** Sarah M. Wilson, Seul Ki Yeon, Xiao-Fang Yang, Ki Duk Park, Rajesh Khanna

**Affiliations:** ^1^Paul and Carole Stark Neurosciences Research Institute, Indiana University School of MedicineIndianapolis, IN, USA; ^2^Center for Neuro-Medicine, Brain Science Institute, Korea Institute of Science and TechnologySeoul, Korea; ^3^Department of Pharmacology, College of Medicine, University of ArizonaTucson, AZ, USA

**Keywords:** CRMP2, GSK3β, CDK5, phosphorylation, mossy fiber sprouting, TIMM staining, epileptogenesis, (*S*)-Lacosamide

## Abstract

Aberrant ion channel function has been heralded as a main underlying mechanism driving epilepsy and its symptoms. However, it has become increasingly clear that treatment strategies targeting voltage-gated sodium or calcium channels merely mask the symptoms of epilepsy without providing disease-modifying benefits. Ion channel function is likely only one important cog in a highly complex machine. Gross morphological changes, such as reactive sprouting and outgrowth, may also play a role in epileptogenesis. Mechanisms responsible for these changes are not well-understood. Here we investigate the potential involvement of the neurite outgrowth-promoting molecule collapsin response mediator protein 2 (CRMP2). CRMP2 activity, in this respect, is regulated by phosphorylation state, where phosphorylation by a variety of kinases, including glycogen synthase kinase 3 β (GSK3β) renders it inactive. Phosphorylation (inactivation) of CRMP2 was decreased at two distinct phases following traumatic brain injury (TBI). While reduced CRMP2 phosphorylation during the early phase was attributed to the inactivation of GSK3β, the sustained decrease in CRMP2 phosphorylation in the late phase appeared to be independent of GSK3β activity. Instead, the reduction in GSK3β-phosphorylated CRMP2 was attributed to a loss of priming by cyclin-dependent kinase 5 (CDK5), which allows for subsequent phosphorylation by GSK3β. Based on the observation that the proportion of active CRMP2 is increased for up to 4 weeks following TBI, it was hypothesized that it may drive neurite outgrowth, and therefore, circuit reorganization during this time. Therefore, a novel small-molecule tool was used to target CRMP2 in an attempt to determine its importance in mossy fiber sprouting following TBI. In this report, we demonstrate novel differential regulation of CRMP2 phosphorylation by GSK3β and CDK5 following TBI.

## Introduction

Precise regulation of voltage- and ligand-gated ion channels is essential for proper function of both the peripheral and central nervous systems. As perturbations to these tightly-controlled systems can result in diverse neuropathologies, regulators of ion channel function have become prime targets for therapeutic intervention. We have previously demonstrated that the intracellular phosphoprotein collapsin response mediator protein 2 (CRMP2) is a positive regulator of both ligand- and voltage-gated calcium channels (Brittain et al., 2009, 2012a) and can be targeted as such to provide therapeutic relief (Brittain et al., 2011a,b; Wilson et al., 2012a). While phosphorylation of CRMP2 increases its interaction with the N-type voltage-gated calcium channel (Brittain et al., 2012b), we recently discovered a novel posttranslational modification (SUMOylation) that negatively impacts CRMP2's ability to enhance calcium influx (Ju et al., 2013). Intriguingly, through SUMOylation, a previously unidentified link between CRMP2 and trafficking of voltage-gated sodium channels was unearthed (Dustrude et al., 2013). CRMP2 is therefore in the unique position of potentially possessing the ability to impact voltage-gated calcium and sodium channels, as well as the ligand-gated N-methyl-D-aspartate (NMDA) receptor. A common pathology to which all three of these channel types are thought to contribute is epilepsy (for review see Ghasemi and Schachter, 2011; Oliva et al., 2012; Siwek et al., 2012).

Nearly 2.3 million people in the United States alone are burdened by epilepsy (CDC, 2012), a neurological condition classified by spontaneously recurring seizures (Goddard et al., 1969), with an estimated 150,000 more diagnosed each year (Hirtz et al., 2007; England et al., 2012). It is estimated that nearly half of epilepsy cases are classified as complex partial seizures, the majority of which originate from foci within the temporal lobe (Hauser and Kurland, 1975; Manford et al., 1992a,b; Larner, 1995; Panayiotopoulos, 2005). In many patients, temporal lobe epilepsy (TLE) is initiated by a traumatic event such as traumatic brain injury (TBI), febrile seizures, status epilepticus (SE), tumors, stroke, or infection (Kharatishvili and Pitkanen, 2010; Yang et al., 2010a; O'Dell et al., 2012). These events are often followed by an asymptomatic latency period lasting upwards of 10 years prior to the development of spontaneous recurring seizures (de Lanerolle et al., 2003; Sharma et al., 2007; Yang et al., 2010a). That these arguably diverse insults can lead to a similar phenotype suggests the possibility of shared epileptogenic mechanisms. The majority of antiepileptic drugs (AEDs) available today target the voltage-gated sodium channel. One of these, Lacosamide (Vimpat^®^) (R-N-benzyl 2-acetamido-3-methoxypropionamide) (*(R)*-LCM), does so through a unique mechanism. Instead of affecting current density or steady-state gating kinetics, (*R*)-LCM selectively enhances sodium channel slow inactivation (Errington et al., 2008). Another characteristic that sets (*R*)-LCM apart from other AEDs is its ability to target CRMP2 (Beyreuther et al., 2007; Park et al., 2009). Notably, expression levels of CRMP2 have been shown to alter the ability of (*R*)-LCM to impact sodium channel function (Wang et al., 2010).

Given CRMP2's remarkable ability to regulate ion channel function, it can be at times difficult to consider its many other functions, particularly those for which it was first identified (i.e., neurite outgrowth and guidance) (Goshima et al., 1995). Importantly, of the myriad of CRMP2 functions, it is the ability to promote neurite outgrowth that is impacted by (*R*)-LCM, not those associated with ion channel function (Wang and Khanna, 2011; Wilson et al., 2012b). CRMP2 promotes neurite outgrowth by two distinct mechanisms: (Brittain et al., 2009) binding and transporting tubulin dimers from the soma to distal projections (Fukata et al., 2002; Kimura et al., 2005) and (Brittain et al., 2012a) stabilizing the growing end of the microtubule by promoting the inherent GTPase activity of tubulin (Chae et al., 2009), the latter of which is impaired by (*R*)-LCM (Wilson et al., 2012b). Aberrant growth and reorganization of neuronal circuits, specifically that of the dentate mossy fibers within the hippocampus, is commonly observed in post-mortem tissue samples from TLE patients and in animal models of the disease (for review see Koyama and Ikegaya, 2004; Sutula, 2004). Notably, CRMP2 was recently suggested to be involved in mossy fiber sprouting in the SE model of TLE (Lee et al., 2012). Under normal conditions, mossy fibers project from the granule cell layer of the dentate gyrus into the CA3 region of the hippocampus where they form synapses with pyramidal cells (Andersen et al., 1969). During TLE, mossy fibers are observed to innervate the inner molecular layer where they synapse onto the dendrites of other dentate granule cells, leading to the formation of recurrent excitatory circuits (Blaabjerg and Zimmer, 2007). To date, the molecular mechanisms contributing to mossy fiber sprouting are relatively unknown. Recent focus has centered on the involvement of growth-factor cascades, particularly that of the tropomycin-related kinase receptor B (TrkB). Activation of the TrkB receptor eventually leads to the phosphorylation and inactivation of glycogen-synthase kinase β (GSK3β) by protein kinase B (Akt) (Cross et al., 1995; Alessi et al., 1996; Bhave et al., 1999). Inactivation of GSK3β has been observed following insults commonly associated with TLE, such as TBI (Shapira et al., 2007; Dash et al., 2011; Zhao et al., 2012), hypoxia-ischemia (Sasaki et al., 2001; Endo et al., 2006; Xiong et al., 2012), and SE (Lee et al., 2012).

The ability of CRMP2 to promote neurite outgrowth is governed by its phosphorylation state. Phosphorylation by a variety of kinases, including GSK3β, renders CRMP2 inactive (Arimura et al., 2000, 2005; Brown et al., 2004; Cole et al., 2004, 2006; Uchida et al., 2005, 2009; Yoshimura et al., 2005; Hou et al., 2009). Specifically, GSK3β phosphorylates CRMP2 at threonines 509, 514, and serine 518, thereby reducing its affinity for tubulin (Yoshimura et al., 2005). As inactivation of GSK3β is observed following TLE-related insults, it is possible that these insults may also lead to a decrease in GSK3β-phosphorylated (inactive) CRMP2, thereby promoting outgrowth. Indeed we have recently demonstrated that targeting CRMP2 with (*R*)-LCM can prevent the increased excitatory connectivity of layer V pyramidal neurons in the neocortical isolation (undercut) model of posttraumatic epileptogenesis (Wilson et al., 2012b). Therefore, if loss of CRMP2 phosphorylation is a contributing factor in mossy fiber sprouting, it may be possible to prevent this reorganization through the use of (*R*)-LCM and its derivatives. In this report we determine if GSK3β phosphorylation of CRMP2 is altered at various stages following a TLE-related insult, as well as, the impact of GSK3β inactivation on CRMP2 function. Ultimately, the role of CRMP2 in mossy fiber sprouting is investigated by selectively targeting CRMP2 function *in vivo*.

Lacosamide was originally discovered to be stereoselective, as much higher concentrations of the (*S*)- configuration are required to halt epileptiform activity both *in vitro* and *in vivo* compared to the (*R*)- configuration (Andurkar et al., 1999; LeTiran et al., 2001; Lees et al., 2006). In this report we employ the use of (*S*)-LCM, which retains the ability to target CRMP2-mediated neurite outgrowth without impacting sodium channel function, to determine if GSK3β phosphorylation of CRMP2 is altered at various stages following a TLE-related insult. Ultimately, the role of CRMP2 in mossy fiber sprouting is investigated by selectively targeting CRMP2 function *in vivo*.

## Materials and methods

### Materials

All reagents were purchased from Sigma (St. Louis, MO, USA) unless otherwise indicated. *(S)*-LCM was provided by the laboratory of Dr. Ki Duk Park, Center for Neuro-Medicine, Brain Science Institute, Korea Institute of Science and Technology. A 100 mM solution was made up in dimethylsulfoxide (DMSO) and stored in small aliquots at −20°C. The final concentration of 200 μM was chosen as it phenocopied the effect of CRMP2 siRNA knockdown (data not shown).

### Cortical neuron culture

Rat cortical neuron cultures were prepared from cortices dissected from embryonic day 19 (E19) rats as described (Goslin and Banker, 1989), with some modifications. Briefly, cortices were dissected out of E19 rats, and cells were dissociated enzymatically and mechanically (trituration through Pasteur pipette) in a Papain solution (12 U/ml; Worthington) containing Leibovitz's L-15 medium (Invitrogen), 0.42 mg/ml cysteine (Sigma), 250 U/ml DNase 1 (type IV; Sigma), 25 mM NaHCO3, penicillin (50 U/ml)/streptomycin (50 μg/ml), 1 mM sodium pyruvate, and 1 mg/ml glucose (Invitrogen). After dissociation, the cells were gently washed by sequential centrifugation in Neurobasal medium containing either 2 mg/ml or 20 mg/ml BSA and Pen/Strep, glucose, pyruvate, and DNase1 (as above) and then plated on poly-D-lysine-coated coverslips or 96-well plates at ~400 cells per mm^2^. Growth media (1 ml/well or 100 μl/well for 12- and 96-well plates, respecitvely) consisted of Neurobasal medium continaing 2% NuSerum, 2% NS21 (Chen et al., 2008), supplemented with penicillin/streptomycin (100 U/ml; 50 μg/ml), 0.1 mM L-Glutamine and 0.4 mML-glutamax (Invitrogen). 5-fluoro-2′-deoxyuridine (1.5 μg/mL) (Sigma) was added 48 h after plating to reduce the number of non-neuronal cells. After 4 days in culture and 2× each week thereon, half of the growth medium was replaced with medium without 5-fluoro-2′-deoxyuridine.

### Neurite outgrowth

Primary cortical neurons plated on 96-well culture plates were transfected via lipofectamine 2000 (Invitrogen) with EGFP at 4 DIV 48 h before imaging with the ImageXpress Micro (Molecular Devices). Immediately prior to imaging, media was exchanged with sterile phosphate buffered saline (PBS). The overexpression of EGFP allowed for visualization of a small percentage of neurons while maintaining optimal cell densities required for survival. EGFP fluorescence was imaged at 4× magnification. To enable laser-based autofocus, laser offset was determined via z-stack. Optimum exposure time was also determined to prevent saturation.

Analysis of neurite outgrowth was completed using a neurite outgrowth analysis protocol within the MetaXpress software (Molecular Devices). Cell soma and processes are detected by defining separate size and fluorescence intensity threshold parameters. Cells were excluded if they were determined not to be neurons based on morphology, if processes extended beyond the image field, or if no processes were longer than 50 μm. The following parameters are recorded and summarized into a final “total outgrowth” parameter: number of processes, number of branches, mean process length, and maximum process length.

### Whole-cell patch-clamp recordings

Whole-cell voltage recordings were performed at RT on primary cultured cortical neurons (7 DIV) using an EPC 10 Amplifier (HEKA Electronics). Electrodes were pulled from thin-walled borosilicate glass capillaries (Warner Instruments) with a P-97 electrode puller (Sutter Instrument) such that the final electrode resistances were 2–3 MΩ when filled with internal solutions. The internal solution for recording Na^+^ currents contained (in mM): 110 CsCl, 5 MgSO_4_, 10 EGTA, 4 ATP Na_2_, and 25 HEPES (pH 7.2, 290–310 mOsmo/L). For recording Na^+^ currents, the external solution contained (in mM): 100 NaCl, 10 tetraethylammonium chloride (TEA-Cl), 1 CaCl_2_, 1 CdCl_2_., 1 MgCl_2_, 10 D-glucose, 4 4-AP, 0.1 NiCl_2_, 10 HEPES (pH 7.3, 310–315 mOsm/L). Whole-cell capacitance and series resistance (70–80%) were compensated with the amplifier. Cells were considered only when the seal resistance was more than 1 GΩ and the series resistance was less than 10 MΩ. Linear leak currents were digitally subtracted by P/4.

### Immunoblot assay and quantification

Protein samples were boiled in Laemmli sample buffer for 5 min and fractionated on 4–15% separating SDS polyacrylamide gels. Apparent molecular weights were determined using broad range standards (Fisher). Following electrophoresis, proteins were transferred to PVDF membranes (Invitrogen) for immunoblotting. Membranes were occasionally stained with ponceau (BioRad) to monitor transfer efficiency. Following transfer, membranes were blocked for 1 h in 5% skim milk powder +0.05% BSA in TBST at RT. Primary antibody incubations [goat anti-rabbit GSK3β and GSK3β pSer9 (Millipore), goat anti-rabbit CRMP2 (Sigma), donkey anti-sheep CRMP2 pThr509/514 (Kinasource), goat-anti-rabbit CRMP2 pSer522 (ECM Biosciences), goat anti-rabbit CDK5 (Cell Signaling), or goat anti-mouse βIII-tubulin (Promega)] were either 2 h at RT or overnight at 4°C. Membranes were extensively washed in TBST and incubated in secondary antibody [goat anti-rabbit, goat anti-mouse, or donkey anti-sheep IgG horseradish peroxidase (HRP)] (G Biosciences) or (goat anti-rabbit, goat anti-mouse IgG dylight 650 or 800 conjugated) (Pierce) (1:15,000). Membranes incubated with HRP-conjugated secondary antibodies were washed extensively in TBST prior to probing with Enhanced Chemiluminescence Western blotting substrate (Fisher) before exposure to photographic film. Blots were exposed for a range of durations to ensure the generation of a print in which the film is not saturated. Membranes incubated with dylight-conjugated secondary antibodies were washed extensively in TBST prior to imaging with the LI-COR Odyssey imaging system. Both images obtained from film and LI-COR were digitized and quantified using Un-Scan-It gel V6.1 scanning software (Silk Scientific Inc., Orem), limiting our analysis to the linear range. Immunoblot images were digitized and analyzed using UnscanIt software, limiting analysis to the digital range. Briefly, densitometric values were obtained for each band and normalized to a loading control (tubulin) for each respective sample. In place of a single background measurement, background values for each band were obtained from an directly adjacent area to avoid issues of background discrepancy. To better allow for direct comparisons, measurements for CDK5- and GSK3β-phosphorylated CRMP2 were compared to values for total CRMP2 and Tubulin from the same immunoblot following a stripping and reprobing procedure. The same method was also applied for comparing phosphorylated-GSK3 to total GSK3 and tubulin. While this method provides a direct comparison between levels of each protein within a single, loaded sample, the stripping procedure does decrease the esthetic quality of the immunoblot.

### Traumatic brain injury

All procedures involving animals were approved by the Institutional Animal Care and Use Committee of Indiana University School of Medicine and were carried out according to NIH guidelines and regulations. Animals were doubly-housed and maintained in a 12 h light/12 h dark cycle environment with access to foor and water *ad libitum*. Adult male Sprague–Dawley rats (275–300 g) were subjected to controlled cortical impact (CCI) injury. Rats were anesthetized with a ketamine/xylazine mixture (80 and 5 mg/kg, respectively) and placed in a stereotaxic frame prior to TBI. Using sterile procedures, the skin was retracted, and a ~4 mm craniotomy was performed ~3 mm lateral to midline and 3 mm posterior to the bregma suture. The skullcap was removed without disruption of the dura. The impacting tip was angled on a medial-lateral plane so that it was perpendicular to the exposed cortical surface. The deformation impact depth was set a 1.5 mm, and the piston velocity was controlled at 3.0 m/s. Following impact, the exposed tissue was covered with bone wax (Henry Schein) and the midline incision was sutured with 5.0 monofilament (Ethicon). Following surgery, animals received a bolus of sterile saline and post-operative analgesic Buprenorphine (0.5 mg/kg). During all surgical procedures and recovery, the core body temperature of the animals were maintained at 36–37°C. Sham animals received the same craniotomy and post-operative care.

### *In vivo* administration of (*S*)-Lacosamide ((*S*)-LCM)

To provide continuous infusion, (*S*)-LCM was delivered via an implanted osmotic mini-pump (Alzet). To compensate for animal growth over the 4-week treatment period, the animals were weighed prior to surgery and the amount of (*S*)-LCM was adjusted to account for the expected weight gain and an infusion rate of 2.5 μl/h to allow for administration of an average of 5 mg/kg per day of (*S*)-LCM or <0.01% DMSO for vehicle. Immediately following CCI surgery, sterile mini-pumps were subcutaneously implanted. An incision was made on the back, between the shoulder blades. A small pocket was created by carefully separating skin from muscle near the incision site. The mini-pump was placed into the pocket and the incision was closed with 5.0 monofilament.

### Tissue processing

For immunoblots: at 24 h or 4 weeks post-TBI, animals were sacrificed and transcardially perfused with 0.1 M phosphate buffer. For perfusion, an incision is made in the left ventricle to allow insertion of a needle attached to a peristaltic pump through the ventricle and into the ascending aorta. The needle was clamped into position and a second incision was made in the right atrium for drainage. Following perfusion, brains were extracted and hippocampi ipsilateral and contralateral to the injury site were dissected, frozen in liquid nitrogen, and stored at −80°C. Prior to immunoblot assay, tissue was thawed and homogenized using a sonicator.

For TIMM staining: at 4 weeks following TBI, animals were sacrificed and transcardially perfused (as previously described) with a sodium sulfide perfusate solution (150 mM NaS_2_, 8.1 mM Na_2_HPO_4_, 1.9 mM NaH_2_PO_4_), followed by 4% paraformaldehyde. Following perfusion, brains were extracted and placed in 4% paraformaldehyde for 24 h at 4°C. Brains were then transferred to 0.1 M phosphate buffer + 30% sucrose for 48 h at 4°C. Tissue was embedded into Optimal Cutting Temperature (OCT) compound (Tissue-Tek) on dry ice. Coronal slices (35 μm thickness) were made on a cryostat (Leica). Slices were mounted onto gelatin-coated microscope slides and stored at −20°C.

### TIMM staining

Tissue sections were allowed to thawed and processed for TIMM staining with the RAPID TIMM Stain Kit (FD Neurotechnologies). Tissue sections were washed in 0.1 M phosphate buffer 3 times, 3 min each and transferred to the TIMM solution (132 mM Citric Acid, 79.5 mM Sodium Citrate, 153.6 mM Hydroquinone, 5 mM AgNO_3_, 30% Gum Arabic), where they were rocked gently in the dark for 45–60 min at 30°C. Sections were then rinsed in ddH_2_O for 3 min in the dark, followed by gently washing them in running water for 30 min to remove excess stain. Sections were dehydrated in 50, 75, and 95% ethanol for 3 min each. Sections were incubated in absolute ethanol 3 times, 3 min each and then cleared in xylene (Fisher) 3 times, 3 min each. Coverslips were added using a resinous mounting medium (Aquamount) (Fisher).

Both low and higher magnification images were obtained using a light microscope (Nikon 90i) and scored by three observers blinded to the conditions, based on the scale originally established by Cavazos et al. (1991). Briefly, the scoring system ranks TIMM staining on a scale of 0–5, with 0 being the absence of TIMM granules within the supragranular region and 5 indicating the existence of a dense band of TIMM granules within the supragranular region, extending into the inner molecular layer. To avoid issues of variance among animals, scores were compared from contralateral and ipsilateral hippocampi from the same animal to yield the difference in TIMM scoring (Ispilateral—Contralateral).

### Data analysis

All data points are shown as mean ± s.e.m. Statistical differences between control and experimental conditions were determined by using ANOVA with a Dunnett's or Tukey's *post-hoc* test or a Student's *t*-test when comparing only two conditions. Values of *p* < 0.05 were judged to be statistically significant.

## Results

In regards to its outgrowth-promoting function, the activity of CRMP2 is regulated by its phosphorylation state. In the unphosphorylated form, CRMP2 is considered active and thereby growth-promoting; however, upon phosphorylation by a variety of kinases, most notably GSK3β, CRMP2 is rendered inactive (Arimura et al., 2000, 2005; Brown et al., 2004; Cole et al., 2004, 2006; Uchida et al., 2005, 2009; Yoshimura et al., 2005; Hou et al., 2009). Tonically active under naïve conditions, GSK3β is inactivated following insults commonly associated with TLE such as TBI (Shapira et al., 2007; Dash et al., 2011; Zhao et al., 2012), hypoxia-ischemia (Sasaki et al., 2001; Endo et al., 2006; Xiong et al., 2012), and SE (Lee et al., 2012). This inactivation of GSK3β may lead to an overall decrease in the level of phosphorylated (inactive) CRMP2, thereby promoting neurite outgrowth.

### GSK3β phosphorylation of CRMP2 under naïve conditions

**Figure 1:**
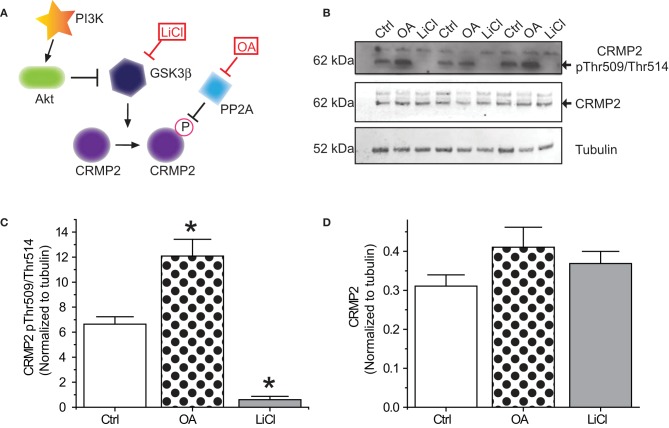
**Phosphorylation of CRMP2 by GSKβ. (A)** Signaling cascade involved in changes in phosphorylation of CRMP2 by GSK3β. Inactivation of GSK3β occurs following activation of Akt or exogenous exposures to lithium chloride (LiCl). Dephosphorylation of CRMP2 by PP2A is prevented by exposure to okadaic acid (OA). **(B)** Levels of GSK3β-phosphorylated CRMP2 and total CRMP2 following 24 h treatment with LiCl (10 mM) or OA (200 nM). **(C,D)** Summary of western blot analysis of GSK3β-phosphorylated CRMP2 and total CRMP2 levels in cortical neurons ± LiCl or OA (^*^p < 0.05 vs. Ctrl, One-Way ANOVA, Dunnet's *post-hoc* analysis) (values represent mean ± s.e.m.) (*n* = 3).

In order for inactivation of GSK3β to impact CRMP2 function, a proportion of CRMP2 must be phosphorylated by GSK3β under normal conditions. Additionally, changes in CRMP2 phosphorylation by GSK3β should occur on a relatively fast time-scale. To determine the extent of GSK3β phosphorylation of CRMP2, primary cultured cortical neurons were exposed to the GSK3β inhibitor Lithium Chloride (LiCl) (10 mM) or the protein phosphatase inhibitor okadaic acid (200 nM) for 18–24 h (Figure 1A). Western blot analysis was performed with an antibody specific to GSK3β-mediated phosphorylation of CRMP2 at Thr509 and Thr514. Importantly, CRMP2 appears to be phosphorylated by GSK3β under naïve/control conditions (Figures 1B,C). Prevention of dephosphorylation by okadaic acid increased CRMP2 phosphorylation by ~2-fold (12.1 ± 1.3) compared to control (6.6 ± 0.6), while LiCl-mediated inhibition of GSK3β resulted in an ~90% loss of CRMP2 phosphorylation (0.6 ± 0.3) (*p* < 0.05) (Figures 1B,C). Levels of total CRMP2 protein remained unchanged (control: 0.31 ± 0.03; okadaic acid: 0.41 ± 0.05; and LiCl: 0.37 ± 0.03) (*p* > 0.05) (Figure 1D). This data suggests that a proportion of CRMP2 is phosphorylated by GSK3β under normal conditions and loss of GSK3β activity dramatically dramatically results in a near-complete loss of phosphorylated CRMP2 within 18–24 h. GSK3β phosphorylation of CRMP2 appears to be dynamically regulated, as evidenced by active dephosphorylation under control conditions.

### GSK3β inhibition increases neurite outgrowth via CRMP2

**Figure 2:**
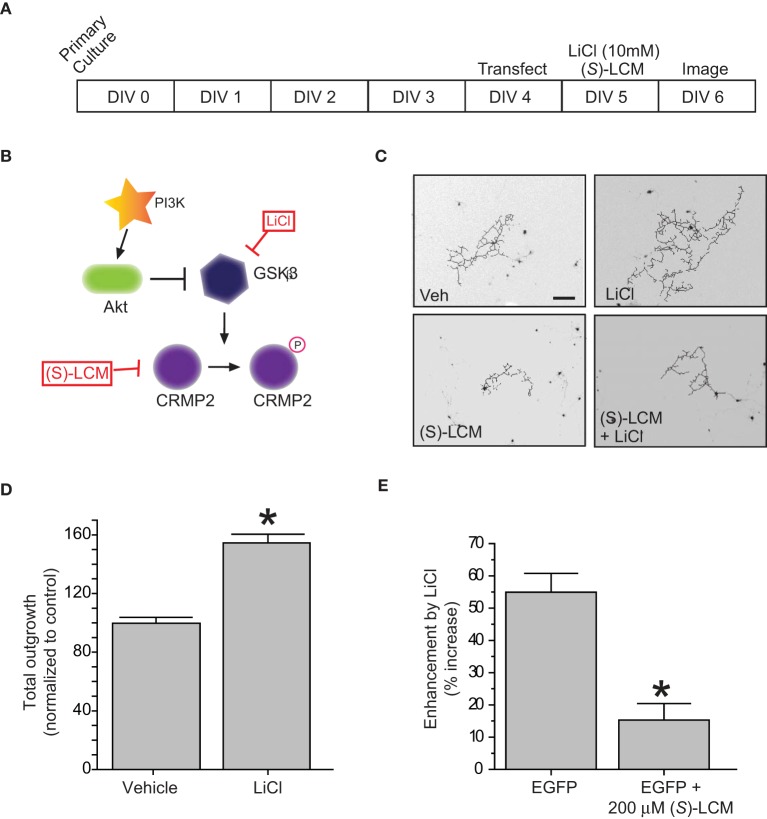
**Inactivation of GSK3β enhances neurite outgrowth in a CRMP2-dependent manner. (A)** Experimental timeline. Cortical neurons were transfected with EGFP at 4 DIV and exposed to vehicle (<0.01% DMSO), LiCl (10 mM), (*S*)-LCM (200 μM), or LiCl + (*S*)-LCM for 24 h starting at 5 DIV and imaged at 6 DIV. **(B)** GSK3β signaling cascade. **(C)** Representative tracings of neurons transfected with EGFP and exposed to LiCl, (*S*)-LCM, or both. (Scale bar = 150 μm). **(D)** Total outgrowth of neurons exposed to LiCl for 24 h (^*^p < 0.05, student's *t*-test) (values represent mean ± s.e.m.). **(E)** Enhancement of outgrowth by LiCl under conditions of (S)-LCM treatment (^*^p < 0.05 vs. EGFP, One-Way ANOVA, Dunnet's *post-hoc* analysis) (values represent mean ± s.e.m.) (*n* = 92–150 cells from 8 separate culture wells).

In regards to its ability to promote neurite outgrowth, phosphorylation by GSK3β effectively inactivates CRMP2 by reducing its affinity for tubulin (Yoshimura et al., 2005). Therefore, inactivation of GSK3β should be growth promoting. To determine if the LiCl-induced loss of GSK3β-phosphorylated CRMP2 directly alters CRMP2-mediated neurite outgrowth within the same timeframe, EGFP-transfected cortical neurons were exposed to lithium chloride for 18–24 h to determine the effect of GSK3β inhibition on neurite outgrowth (Figure 2A). Immediately following exposure, neurons were imaged using the ImageXpress Micro system and neurite outgrowth was determined via the MetaXpress software system. As expected, inhibition of GSK3β increased total outgrowth (154.5 ± 5.9) compared to controls (99.8 ± 4.0) (*p* < 0.05) (Figures 2B–D). We had previously demonstrated that (*S*)-LCM, the inactive enantiomer of (*R*)-LCM, retains the ability to target CRMP2 function and can therefore be used in place of knockdown strategies to determine the role of CRMP2 in a particular process (Wilson and Khanna, unpublished). To ensure that the LiCl-induced increase in outgrowth was in fact due to changes in CRMP2 activity, the experiment was repeated in the presence of *(S)*-LCM (200 μM). As *(S)*-LCM alone decreases outgrowth, it was included in both LiCl-treated and control conditions. Without (*S*)-LCM, LiCl increased neurite outgrowth by 54.9 ± 5.9% (Figure 2D). In contrast, in the presence of (*S*)-LCM, LiCl increased neurite outgrowth by 15.3 ± 5.1% (*p* < 0.05) (Figures 2D,E).

### (*S*)-LCM does not target voltage-gated sodium channels

**Figure 3:**
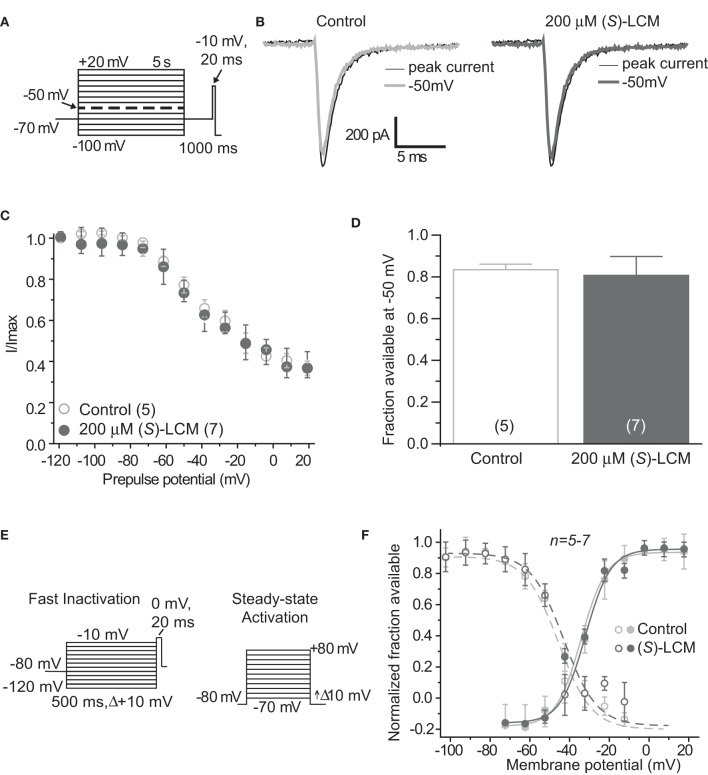
**(*S*)-Lacosamide ((*S*)-LCM) does not affect biophysical properties of voltage-gated sodium currents in cortical neurons. (A)** Voltage protocol for slow inactivation. **(B)** Representative current traces from cortical neurons in the absence (control, 0.1% DMSO) or presence of 200 μM (S)-LCM. The thin black and thick gray traces represent the currents evoked at −100 and −50 mV, respectively (also highlighted in the voltage protocol as a dashed thick line). **(C)** Summary of steady-state slow activation curves for neurons treated with DMSO (control) or 200 μM (S)-LCM. No drug-induced slow inactivation was evident at voltages more depolarizing that −80 mV in neurons treated with (*S*)-LCM. **(D)** Summary of the fraction of current available at −50 mV for neurons treated with DMSO (control) or 200 μM (S)-LCM (*p* > 0.05, Student's *t*-test). **(E)** Voltage protocol for fast inactivation (left) and activation (right). **(F)** Representative Boltzmann fits for steady-state fast inactivation and activation for neurons treated with 0.1% DMSO (control) and or 200 μM (S)-LCM are shown. Values for V1/2, the voltage of half-maximal inactivation and activation and the slope factors (k) were derived from Boltzmann distribution fits to the individual recordings and averaged to determine the mean (±s.e.m.) voltage dependence of steady-state inactivation and activation, respectively. There were no differences between control and drug for any of the parameters tested (*p* > 0.05, One-Way ANOVA). Data are from 5 to 7 cells per condition.

It is essential to verify that (*S*)-LCM is unable to impact voltage-gated sodium channels in this system. Therefore, sodium channel slow inactivation of cultured cortical neurons was determined by holding cells at −70 mV, conditioning to potentials ranging from −100 to +20 mV (in +10 mV increments) for 5 s, moved to a hyperpolarizing pulse of −120 mV for 150 ms to allow fast-inactivated channels to recover, and a single depolarizing pulse to 0 mV was applied for 15 ms to determine the fraction of channels available (Figure 3A). The addition of 200 μM (*S*)-LCM did not alter the onset or extent of slow inactivation (Figures 3B–D). We next determined the effect of (*S*)-LCM on fast inactivation and steady-state activation. For fast inactivation, cells were held at −80 mV, conditioned to potentials ranging from −120 to −10 mV (in +10 mV increments) for 500 ms and the fraction of available current was determined by a 20 ms test pulse at 0 mV (Figure 3E). For steady-state activation, cells were held at −80 mV and current was measured at potentials ranging from −70 to +80 mV (in +10 mV increments) for 500 ms (Figure 3E). Neither fast inactivation nor steady-state inactivation was effected by (*S*)-LCM (Figure 3F).

### Loss of CRMP2 phosphorylation following TBI

**Figure 4:**
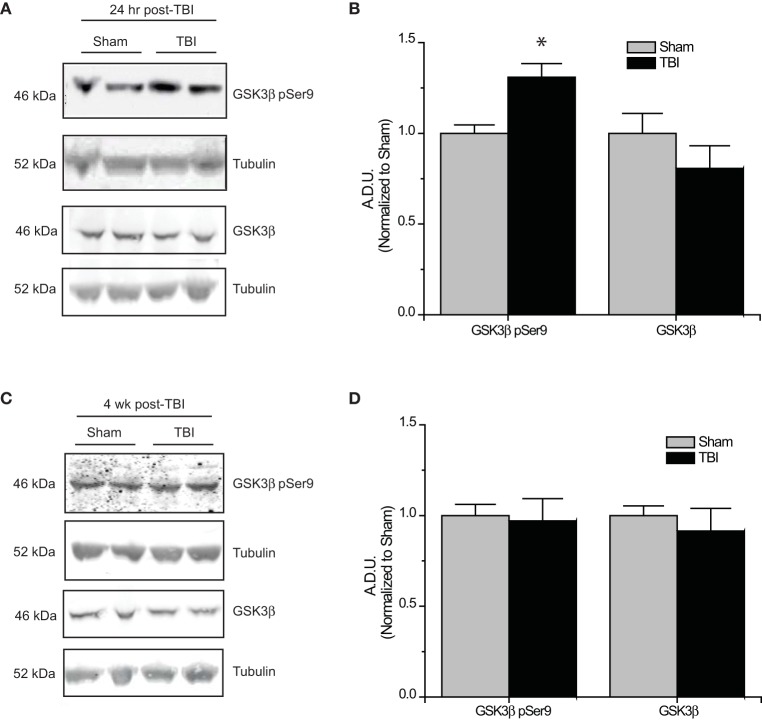
**Changes in GSK3β phosphorylation following TBI. (A)** Western blots of phosphorylated and total GSK3β from hippocampal tissue 24 h following TBI. **(B)** Summary of GSK3β pSer9 and total GSK3β levels 24 h following TBI. [Data is depicted as arbitrary densitometric units (A.D.U.)]. **(C)** Western blots of phosphorylated and total GSK3β from hippocampal tissue 4 weeks following TBI. **(D)** Summary of GSK3β pSer9 and total GSK3β levels 4 weeks following TBI (^*^p < 0.05 vs. sham, Student's *t*-test) (values represent mean ± s.e.m.) (*n* = 4–5).

The development of TLE following TBI, accounts for 20% of symptomatic epilepsy (Agrawal et al., 2006). Evidence of increased Akt activation within the hippocampus as well as other regions has been observed following TBI (Zhang et al., 2006; Zhao et al., 2012). Corresponding to changes in Akt activity, levels of phosphorylated (inactive) GSK3β are also increased following TBI (Shapira et al., 2007; Dash et al., 2011; Zhao et al., 2012). To determine if changes in CRMP2 phosphorylation could be the driving force behind the increased neurite outgrowth in the hippocampus following TLE-related insults, hippocampal tissue was collected at both early (24 h) and late (4 weeks) phases following TBI in adult male rats. Consistent with previous reports, levels of Ser9-phosphorylated (inactivated) GSK3β were increased in the early phase following TBI (1.31 ± 0.08) compared to sham controls (1.00 ± 0.05) (*p* < 0.05) (Figures 4A,B). Importantly, total expression of GSK3β remained unchanged [(1.00 ± 0.11) vs. (0.81 ± 0.12)] (*p* > 0.05) (Figures 4A,B). The increase in GSK3β phosphorylation appeared to be transient, as levels did not differ at 4 weeks following TBI (0.97 ± 0.12) compared to sham controls (1.00 ± 0.06) (*p* > 0.05) (Figures 4C,D).

**Figure 5:**
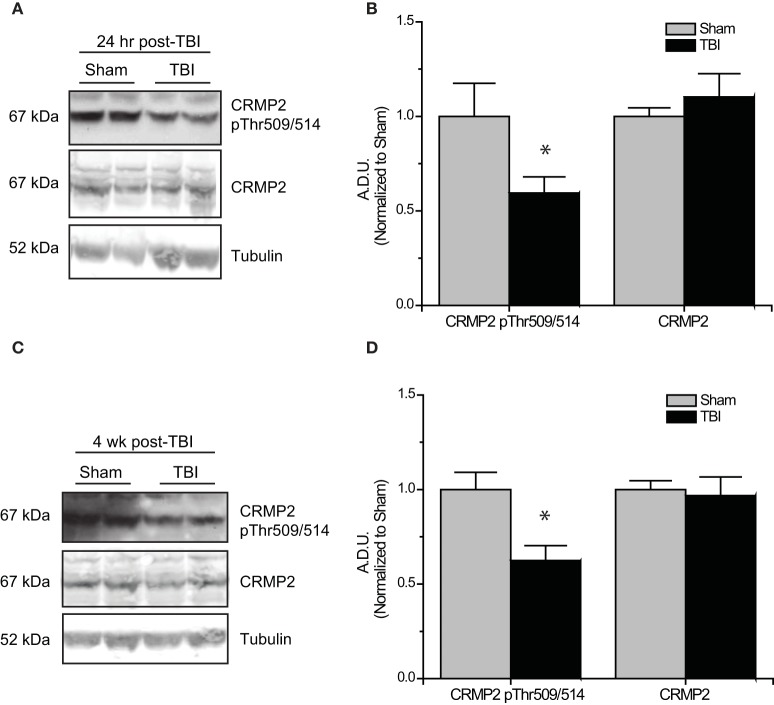
**Changes in CRMP2 phosphorylation by GSK3β following TBI. (A)** Western blots of GSK3β-phosphorylated and total CRMP2 from hippocampal tissue 24 h following TBI. **(B)** Summary of CRMP2 pThr509/514 and total CRMP2 levels 24 h following TBI (raw data represents protein of interest normalized to tubulin and further normalized to sham to allow for easy comparison) [data is represented as arbitrary densitometric units (A.D.U)]. **(C)** Western blots of GSK3β-phosphorylated and total CRMP2 from hippocampal tissue 4 weeks following TBI. **(D)** Summary of CRMP2 pThr509/514 and total CRMP2 levels 4 weeks following TBI. Data was normalized to sham conditions for ease of comparison. (^*^p < 0.05 vs. sham, Student's *t*-test) (values represent mean ± s.e.m. from *n* = 4–5).

Subsequent to the observed inactivation of GSK3β, levels of GSK3β-phosphorylated CRMP2 were reduced in the early phase following TBI (0.52 ± 0.07) compared to sham controls (1.00 ± 0.13) (*p* < 0.05) (Figures 5A,B). No change in total CRMP2 expression was observed [(1.10 ± 0.12) vs. (1.00 ± 0.04)] (*p* > 0.05) (Figures 5A,B). Despite the observed transience of GSK3β inactivation, levels of GSK3β-phosphorylated CRMP2 remained reduced in the late phase following TBI (0.62 ± 0.08) compared to sham controls (1.00 ± 0.09) (*p* < 0.05) (Figures 5C,D). These results suggest that there is an increased level of active (unphosphorylated) CRMP2 in both the early and late phases following TBI. Interestingly, while the decrease in CRMP2 phosphorylation at 24 h post-TBI is directly correlated with an inactivation of GSK3β, the sustained decrease observed at 4 weeks post-TBI appears to be independent of changes in GSK3β activity.

### “Primed” CRMP2 is decreased in the late, but not early phase following TBI

**Figure 6:**
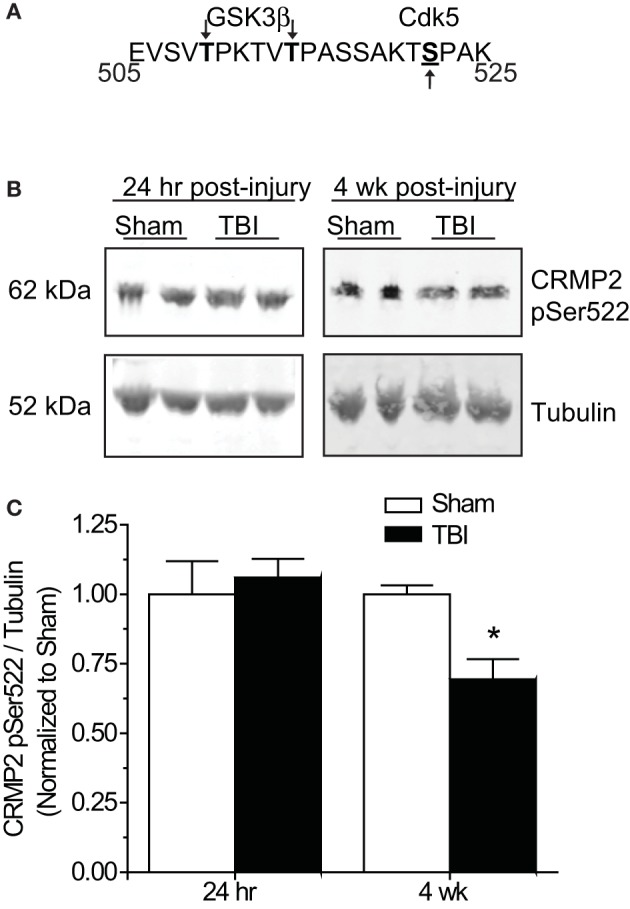
**Changes in CRMP2 phosphorylation by CDK5 following TBI. (A)** Schematic of GSK3β and Cdk5 phosphorylation sites within the rat CRMP2 sequence. Numbers represent amino acid residues within the CRMP2 sequence. **(B)** Western blots of CDK5-phosphorylared CRMP2 from hippocampal tissue 24 h and 4 weeks following TBI. **(C)** Summary of CRMP2 pSer522 levels at both 24 h and 4 weeks following TBI. For ease of comparison, data was normalized to the sham conditions. Levels of CRMP2 pSer522 were decreased at 4 weeks, but not 24 h following TBI. (^*^p < 0.05, student's *t*-test) (values represent mean ± s.e.m. from *n* = 4–5).

Phosphorylation of GSK3β is not the only avenue through which phosphorylation of its substrates is regulated. GSK3β substrate recognition can be complex, as there is no strict consensus motif, often requiring prior phosphorylation (priming) at a serine slightly c-terminal to the GSK3β site(s) (DePaoli-Roach, 1984; Fiol et al., 1988). This type of hierarchical phosphorylation allows for complex regulation at multiple levels. In the case of CRMP2, it must first be phosphorylated at serine 522 by the serine/threonine kinase cyclin-dependent protein kinase 5 (CDK5) in order to be phosphorylated by GSK3β (Yoshimura et al., 2005; Cole et al., 2006) (Figure 6A). Sequential phosphorylation of CRMP2 by CDK5 and GSK3β has been demonstrated for many facets of CRMP2 function, most importantly, neurite outgrowth and growth cone collapse (Brown et al., 2004; Uchida et al., 2005). As decreased levels of GSK3β-phosphorylated CRMP2 were observed in the late phase following TBI that were not secondary to changes in GSK3β expression or activity, it is possible that the changes in levels of GSK3β-phosphorylated CRMP2 may be attributed to a decrease in phosphorylation by CDK5. Therefore, levels of CDK5-phosphorylated CRMP2 were determined from hippocampal tissue collected at early (24 h) and late (4 weeks) time points following TBI. Notably, CDK5 phosphorylation of CRMP2 at 24 h following TBI did not differ from sham controls [(1.05 ± 0.07) vs. (1.00 ± 0.12)] (*p* > 0.05) (Figures 6B,C). However, at 4 weeks following injury, levels of CDK5-phosphorylated CRMP2 were decreased (0.69 ± 0.07) compared to sham controls (1.00 ± 0.03) (*p* < 0.05) (Figures 6B,C). These results suggest that CRMP2 is differentially regulated during early and late phases following injury. While a loss of GSK3β activity accounts for decreases in CRMP2 phosphorylation immediately following injury, the same phenotype during later phases is attributed to a loss of priming by CDK5.

### Effects of targeting CRMP2 *in vivo* on mossy fiber sprouting

**Figure 7:**
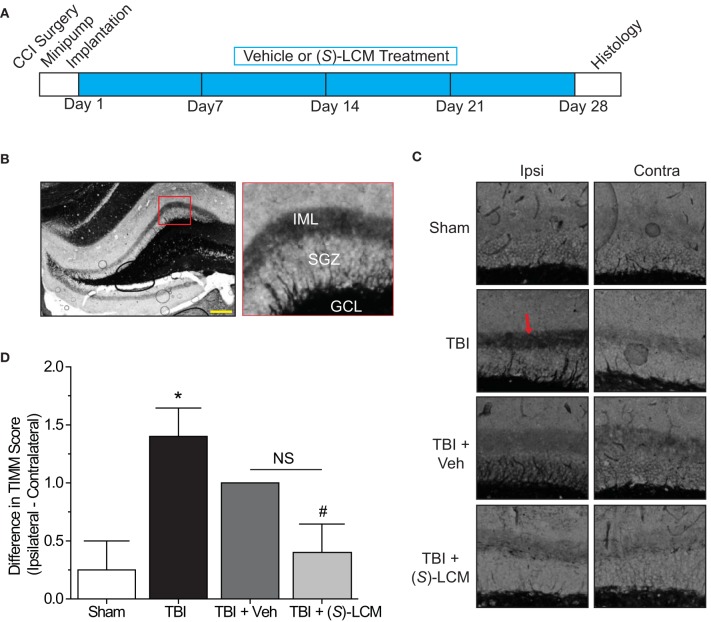
**Effects of targeting CRMP2 *in vivo* on mossy fiber sprouting. (A)** Timeline of experimental design. Animals received either controlled cortical impact or sham (craniotomy) surgery. Immediately following surgery, animals were implanted with osmotic mini-pumps containing either vehicle or (*S*)-LCM to be continuously infused at <0.01% DMSO and ~5 mg/kg per day. Following 4 weeks of treatment, tissue samples were prepared for histology. **(B)** Representative low-magnification image of a TIMM-stained coronal section. Red box depicts region quantified for the extent of mossy fiber sprouting into the inner molecular layer. **(C)** Representative 10×-magnification images of ipsilateral and contralateral TIMM-stained hippocampi. TBI led to a dense laminar band of TIMM reactivity within the supragranular zone extending to the inner molecular layer (*red arrow*). **(D)** Summary of TIMM scores from animals exposed to sham or TBI surgery ± vehicle or (*S*)-LCM. To minimize the impact of variance among animals, data is represented as the difference in TIMM score between ipsilateral and contralateral hippocampi from the same animal. (*S*)-LCM treatment prevented the TBI-induced increase in mossy fiber sprouting, however did not differ from vehicle. (^*^p < 0.05 vs. sham; ^#^*p* < 0.05 vs. TBI; One-Way ANOVA, Tukey's *post-hoc* analysis) (values represent mean ± s.e.m. from *n* = 4–5). Scale bar = 250 μm.

As changes in CRMP2 phosphorylation, and presumably activity, are observed within the hippocampus at both early and late time points following TBI, CRMP2 may be involved in both the induction and maintenance of mossy fiber sprouting following injury. To determine the importance of CRMP2 in this phenomenon, osmotic minipumps containing *(S)*-LCM (140 mg/kg) were implanted (subcutaneously) immediately following TBI surgery in adult male rats. This method allowed for continuous delivery of ~5 mg/kg *(S)*-LCM per day (~0.21 mg/kg per hour) over the course of 4 weeks (Figure 7A). The extent of aberrant mossy fiber sprouting is easily identified due to the high amount of chelatable zinc within mossy fibers which can be visualized via a silver-sulfide staining method (TIMM staining) (Timm, 1958; Zimmer, 1973). Therefore, at the cessation of treatment, bilateral hippocampal tissue was obtained and processed for TIMM staining, to reveal the extent of mossy fiber sprouting within the inner molecular layer of the hippocampus.

As expected, TBI led to increased TIMM differences (1.40 ± 0.25) compared to sham controls (0.25 ± 0.25) (*p* < 0.05) (Figures 7B–D). Importantly, differences in TIMM scores did not differ between sham (0.25 ± 0.25) and naïve animals (0.00 ± 0.32) (*p* > 0.05). Intriguingly, *(S)*-LCM treatment prevented the TBI-induced increase in TIMM differences (0.40 ± 0.25) compared to animals receiving TBI alone (1.40 ± 0.25) (*p* < 0.05) (Figures 7B–D). However, the changes in TIMM scores following TBI did not differ between animals receiving *(S)*-LCM (0.40 ± 0.25) and vehicle (~0.01%DMSO) (1.00 ± 0.00) (*p* > 0.05) (Figures 7C–D). Therefore, it cannot definitively be concluded that CRMP2 is necessary for mossy fiber sprouting following TBI.

## Discussion

In order for changes in GSK3β activity to impact CRMP2 function, a balance of GSK3β-phosphorylated and unphosphorylated CRMP2 must be present. Importantly, GSK3β phosphorylation of CRMP2 appears to be dynamically regulated in naïve neurons, as inhibition of GSK3β led to an almost complete loss of phosphorylation within 24 h. Additionally, the increase in phosphorylation following okadaic acid exposure provides evidence for active dephosphorylation. Therefore, changes in GSK3β activity can directly impact CRMP2 function. Indeed, inhibition of GSK3β led to increases in neurite outgrowth in a CRMP2-dependent manner. Given that inactivation of GSK3β has previously been demonstrated following TBI, decreased phosphorylation of CRMP2 may account for the changes in neurite elongation and branching observed within the hippocampus. Our findings indicate that TBI leads to decreased GSK3β phosphorylation of CRMP2 at both 24 h and 4 weeks post-injury. As mossy fiber sprouting is considered a progressive process, the maintained loss of phosphorylation throughout later phases following injury is an important finding. In contrast to early phases following TBI, the loss of GSK3β phosphorylation at 4 weeks post-injury is likely not attributed to a prolonged inactivation of GSK3β. In fact, previous reports suggest that levels of Akt-phosphorylated GSK3β return to baseline within 14 days (Dash et al., 2011). These findings suggest that while CRMP2 may play an integral role in promoting neurite outgrowth both immediately following injury as well as in later phases, the mechanisms underlying the increase in CRMP2 activity during these phases may differ. Indeed, our results revealed that the decrease in GSK3β-phosphorylated CRMP2 during later phases following injury was in fact attributed to a decrease in priming by CDK5.

Mossy fiber sprouting in TLE can likely be divided into 2 distinct phases: the induction phase, during which sprouting and outgrowth are attributed directly to the precipitating insult such as TBI, hypoxia-ischemia, or SE, and the maintenance phase (Sutula, 2004; Pitkänen and Lukasiuk, 2009). Therefore, early events following injury, such as inactivation of GSK3β, can be attributed to injury-induced mechanisms (i.e., activation of pro-survival signaling pathways). The latter phase involves processes secondary to the original insult such as hyperexcitability and network synchronization. Interestingly, we have previously demonstrated that CDK5 priming of CRMP2 is decreased in response to prolonged neuronal activity and that the loss of priming is directly translated into a reduction in GSK3β-phosphorylated CRMP2 (Wilson and Khanna, unpublished). As TBI can lead to progressive hyperexcitability (Yang et al., 2010b), the sustained loss of CRMP2 phosphorylation is potentially due to progressive changes in neuronal function which are secondary to the precipitating injury, such as activity-driven changes in CDK5 function.

Overall, phosphorylation of CRMP2 appears to be differentially regulated through both induction (early) and maintenance (late) phases following TBI. As such, targeting CRMP2-mediated neurite outgrowth throughout these stages may be sufficient to attenuate the progression of mossy fiber sprouting. Indeed, the extent of mossy fiber sprouting in animals that had received continuous administration of *(S)*-LCM following TBI was markedly decreased compared to untreated animals. However, the trending effect of vehicle administration is a confounding factor that prevents a definitive conclusion from being drawn. Changes in hippocampal cell death likely do not account for the observed changes in TIMM staining, as dentate granule cell survival is typically not impacted by moderate TBI (Lowenstein et al., 1992; Grady et al., 2003). Furthermore, (S)-LCM is unlikely to affect hippocampal cell death as a recent study demonstrated that the parent compound (R)-LCM does not alter neuronal survival following TBI (Pitkanen et al., 2014). The lack of significant separation between *(S)*-LCM- and vehicle-treated groups may be a result of the nature of administration, despite the previous success observed with infusion of *(R)*-LCM in a separate study (Licko et al., 2013). While continuous subcutaneous infusion was considered to be preferable over daily intraperitoneal injections, it is possible that inflammation at the implantation site may have been a factor.

**Figure 8:**
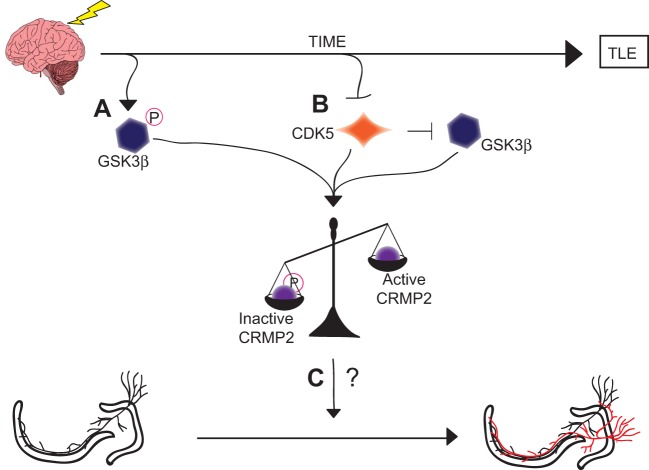
**Graphical summary of findings. (A)** GSK3β is phosphorylated and thereby inactivated in the early phases following injury. This inactivation leads to decreased amounts of phosphorylated (inactive) CRMP2. **(B)** CDK5 phosphorylation of CRMP2 is decreased in the later phases following injury. This decrease in phosphorylation also indirectly reduces levels of GSK3β-phosphorylated CRMP2 through a loss of priming, resulting in an overall increase in the proportion of active CRMP2. **(C)** Despite the sustained increase in unphosphorylated (active) CRMP2 in the hippocampus, the role of CRMP2 in aberrant mossy fiber sprouting following injury remains unclear, as denoted by the “?.”

Although the exact role of CRMP2 in mossy fiber sprouting has not yet been determined, it is possible that the loss of GSK3β phosphorylation immediately following injury contributes to the induction of mossy fiber sprouting while the loss of priming by CDK5 in later phases contributes to the maintenance of mossy fiber sprouting. It is of great interest that these mechanistically distinct events culminate in a similar end-point: an increase in the amount of active CRMP2 (Figure 8). The reduction in mossy fiber sprouting in *(S)*-LCM-treated animals suggests, at the very least, that CRMP2 may be one factor involved in initiation and progression of mossy fiber sprouting.

## Authors and contributors

Participated in Research Design—Sarah M. Wilson, Rajesh Khanna. Conducted Experiments—Sarah M. Wilson, Seul Ki Yeon, Xiao-Fang Yang. Performed Data Analysis—Sarah M. Wilson, Seul Ki Yeon, Ki Duk Park, Xiao-Fang Yang. Wrote the Manuscript—Sarah M. Wilson, Rajesh Khanna.

### Conflict of interest statement

The authors declare that the research was conducted in the absence of any commercial or financial relationships that could be construed as a potential conflict of interest.
